# Comparative study of gut microbiota reveals the adaptive strategies of gibbons living in suboptimal habitats

**DOI:** 10.1038/s41522-025-00653-6

**Published:** 2025-02-14

**Authors:** Li-Ying Lan, Tai-Cong Liu, Shao-Ming Gao, Qi Li, Li Yang, Han-Lan Fei, Xu-Kai Zhong, Yu-Xin Wang, Chang-Yue Zhu, Christoph Abel, Peter M. Kappeler, Li-Nan Huang, Peng-Fei Fan

**Affiliations:** 1 School of Life Sciences, Sun Yat-sen University, Guangzhou, P.R. China; 2 Behavioral Ecology and Sociobiology Unit, German Primate Center, Leibniz Institute for Primate Research, Göttingen, Germany; 3 School of Environmental Science and Engineering, Sun Yat-sen University, Guangzhou, P.R. China; 4 College of Life Sciences, China West Normal University, Nanchong, P.R. China; 5 Department of Sociobiology/Anthropology, Johann-Friedrich-Blumenbach Institute of Zoology and Anthropology, University of Göttingen, Göttingen, Germany

**Keywords:** Microbiome, Bacteria, Microbial ecology

## Abstract

Wild animals face numerous challenges in less ideal habitats, including the lack of food as well as changes in diet. Understanding how the gut microbiomes of wild animals adapt to changes in food resources within suboptimal habitats is critical for their survival. Therefore, we conducted a longitudinal sampling of three gibbon species living in high-quality (*Nomascus hainanus*) and suboptimal (*Nomascus concolor* and *Hoolock tianxing*) habitats to address the dynamics of gut microbiome assembly over one year. The three gibbon species exhibited significantly different gut microbial diversity and composition. *N. hainanus* showed the lowest alpha diversity and highest nestedness, suggesting a more specialized and potentially stable microbial community in terms of composition, while *H. tianxing* displayed high species turnover and low nestedness, reflecting a more dynamic microbial ecosystem, which may indicate greater sensitivity to environmental changes or a flexible response to habitat variability. The gut microbial community of *N. concolor* was influenced by homogeneous selection in the deterministic process, primarily driven by Prevotellaceae. In contrast, the gut microbial communities of *H. tianxing* and *N. hainanus* were influenced by dispersal limitation in the stochastic process, driven by Acholeplasmataceae and Fibrobacterota, respectively. Further, the microbial response patterns to leaf feeding in *N. hainanus* differed from those of the other two gibbon species. In conclusion, this first cross-species comparative study provides initial insights into the different ecological adaptive strategies of gut microbiomes from a point of community assembly, which could contribute to the long-term conservation of wild primates.

**Figure:**
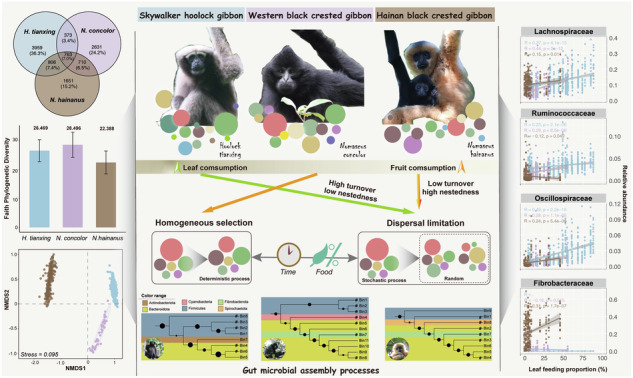
In this study, we conducted longitudinal sampling of three gibbon species living in high-quality (*Nomascus hainanus*) and suboptimal (*Nomascus concolor* and *Hoolock tianxing*) habitats to address the dynamics of gut microbiome (composition, alpha diversity, beta diversity and assembly process) over one year.

In this study, we conducted longitudinal sampling of three gibbon species living in high-quality (*Nomascus hainanus*) and suboptimal (*Nomascus concolor* and *Hoolock tianxing*) habitats to address the dynamics of gut microbiome (composition, alpha diversity, beta diversity and assembly process) over one year.

## Introduction

Large remnant populations of endangered animals have migrated to suboptimal habitats due to human activities, further resulting in population decline and global biodiversity loss^1–3^. In suboptimal habitats, one of the key behavioral adaptations of wildlife is to consume large amounts of alternative foods to cope with the nutritional demands of food shortages in cold seasons. As the digestive system is unable to keep up with the rapidly changing environment evolutionarily, the fast response of the gut microbiota and the associated metabolic activity may be a key mechanism for adapting the host to dietary transitions^4^. Recent research has emphasized the importance of host-associated microbes to the well-being and conservation of endangered animals^5–7^. However, those studies were either single-species studies or comparisons of completely different diets, neglecting quantitative diet data and comparisons in different suboptimal environments across species. In addition, many studies have emphasized the great significance of conducting research using long-term dietary monitoring to investigate temporal gut microbiome dynamics due to the high flexibility of gut microbiota^8–10^. Therefore, longitudinal and cross-species comparative studies on gut microbes could give us an opportunity to understand the threshold of adaptation to suboptimal environments in wildlife.

Recently, more evidence has revealed that higher gut microbial alpha diversity (local species richness) is associated with a positive effect on animals, due to increased pathogen resistance in the host^11^, more diverse functionality^12,13^, and greater functional redundancy^14,15^. However, little attention has been paid to gut microbial beta diversity and microbial assembly processes. Beta diversity describes the variation in species composition among sites and can result from species replacement between sites (turnover) and species loss from site to site (nestedness)^16^. Beta diversity is especially important in the context of ecosystem multifunctionality^17^, while the microbial assembly process (the presence and abundance of species) can influence microbial diversity and composition, with downstream impacts on the function of the system^18^. The microbial assembly process falls into two predominant categories^19–21^: (i) stochastic processes, in which species are independent of respective niches in habitats^22,23^ and (ii) deterministic processes, in which the establishment and success of species in an environment largely rely on their respective niches and ecological fitness^20^. If the environmental filters are too weak to cause selection, stochastic community assembly will become the main process. However, stronger environmental filters will lead to heterogeneous and homogeneous selection in deterministic processes^19^. Exploring gut microbial assembly processes could effectively reflect the response of the microbial community to the internal gut environment and external environment of the host. Currently, microbial assembly processes are widely used in ecology, especially for studying microbial communities, like in the ocean and soil^24–26^. Even though they provide a good theoretical basis for exploring the dynamic microbial communities, they are rarely used in wildlife gut microbial research due to the late start of this discipline and the difficulties of long-term sampling.

Gibbons (family Hylobatidae) are arboreal animals and often considered flagship species in forest ecosystems^27^. Currently, population numbers of all 20 gibbon species are declining, with five critically endangered, fourteen endangered, and one vulnerable species^28^. The potential habitats of gibbons have decreased significantly due to forest fragmentation. As a result, many gibbon species have migrated to habitats, previously considered suboptimal, in northern areas or higher altitudes^29,30^. During long-term field studies, researchers found that northern gibbons (some gibbons involved in this study) are facing bigger survival challenges than those in subtropical habitats, including food shortages and low-temperature conditions in certain seasons^4,31,32^. For example, they feed heavily on alternative foods (leaves) during periods of high-energy food (fruit) deprivation at high altitudes^33,34^, even though they are more likely to feed on fruit in fruit-rich habitats or seasons^31^. Leaves are an abundant resource in the forest and are generally considered as low-quality food^35^. Unlike some Colobinae primates, gibbons do not have well-developed stomachs or complex hindgut structures to help them ferment and digest fiber in leaves as frugivorous animals^36,37^. Thus, gibbons are an ideal model to study how gut microbes in endangered wildlife respond to host adaptations to suboptimal habitats, which are closely linked to their survival and conservation.

Here, we overcame the difficulties of dietary data quantification, individual recognition, and long-term sampling, demonstrating in detail the dynamics of the gut microbial assembly across different seasons and gibbon species living in high-quality or suboptimal habitats. This is the first study across species of quantitative descriptions of seasonal changes in gut microbes. We analyzed how the gut microbes (alpha diversity, beta diversity and assembly process) respond to seasonal dietary changes in different habitats. Our study sought to resolve (1) whether and how the effects of diet on the gut microbial community assembly process differ between gibbon species and seasons and (2) what microbial taxa respond to dietary changes and contribute to the changes in microbial community assembly.

## Results

### Comparisons of microbial taxonomic membership across gibbons

**Fig. 1:**
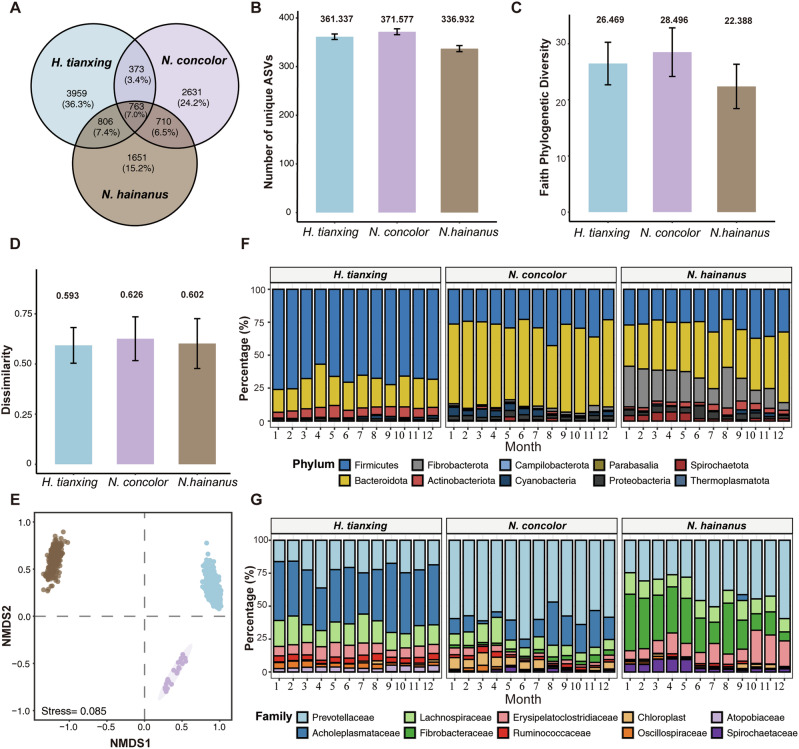
Comparative analysis of microbial diversity in three gibbon species. **A** the unique and shared amplicon sequence variants (ASVs) among gibbon species. **B** number of unique ASVs per sample of all gibbon species. **C** alpha diversity: faith’s phylogenetic diversity index. **D** beta diversity of community dissimilarity. **E** gut bacterial structure in gibbons via NMDS. **F** microbial membership at phylum level. **G** microbial membership at family level.

The taxonomic analysis of microbes from 947 samples yielded 16 phyla, 23 classes, 44 orders, 65 families, 137 genera, and 10893 ASVs, among which 763 ASVs were shared by the three gibbon species. *H. tianxing* had the highest proportion of unique ASVs (*n* = 3959), followed by *N. concolor* (*n* = 2631) and *N. hainanus* (*n* = 1651) (Fig. 1A). Accounting for the number of ASVs across fecal samples, the three gibbons displayed similar number of ASVs per sample (Fig. 1B). Significant differences in community diversity and structure of gut microbes were detected among the three species (Fig. 1C, D, Kruskal–Wallis test: *P* < 0.001; Fig. 1E, PERMANOVA test: all pairs *P* < 0.001), with *N. concolor* having the highest and *N. hainanus* having the lowest alpha diversity (Faith’s phylogenetic diversity, Fig. 1C).

At the phylum level (Fig. 1F), the most abundant gut bacteria of *H. tianxing*, *N. concolor* and *N. hainanus* were Firmicutes (mean relative abundance: 68%, 62%, and 28.7%, respectively) and Bacteroidota (22.3%, 27.2%, and 38.7%, respectively). In addition, *N. hainanus* had a high proportion of Fibrobacterota (20.3%). At the family level (Fig. 1G), the abundant bacterial families differed among the three gibbons. In *H. tianxing*, the most abundant bacteria were Acholeplasmataceae (33.1%), Prevotellaceae (18.0%) and Lachnospiraceae (12.8%), which accounted for about 64% of the detectable reads in all samples (Fig. 1G). In *N. concolor*, the most abundant bacterial families were Prevotellaceae (51.1%), Acholeplasmataceae (12.8%) and Lachnospiraceae (7.63%), which accounted for about 71% of the detectable reads in all samples (Fig. 1G). In *N. hainanus*, the most abundant bacterial families were Prevotellaceae (31.1%), Fibrobacteraceae (22.5%), Erysipelatoclostridiaceae (11.0%) and Lachnospiraceae (10.3%), which accounted for about 75% of the detectable reads in all samples (Fig. 1G).

### Comparisons of seasonal and dietary influences on gibbon gut microbial dynamics

**Fig. 2:**
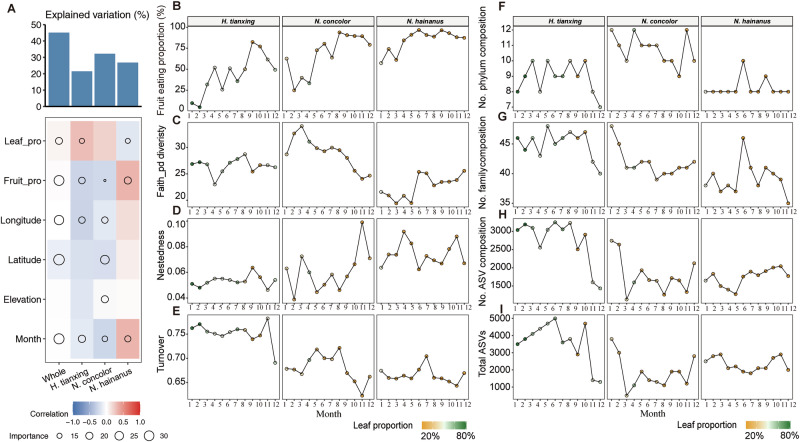
Seasonal and dietary influences on gut microbial dynamics in three gibbon species. **A** Impact of season and diet on gut microbiome (random forest model). **B** Seasonal fruit consumption patterns. **C** Alpha diversity: faith’s phylogenetic diversity index over time. **D** Beta diversity: nestedness over time. **E** Beta diversity: species turnover over time. **F** Number of unique phyla over time in each gibbon species. **G** Number of unique bacterial families over time in each gibbon species. **H** Number of unique ASVs over time. **I** Total ASVs over time.

Analyzing the temporal dynamics, the random forest results showed that diet (leaf-feeding proportion or fruit feeding proportion) and seasonal variation (month) across all factors contributed most toward explaining variation in bacterial alpha diversity of all gibbon species (Fig. 2A and Supplementary Table 1). This result was also confirmed by Spearman’s rank correlation (Supplementary Fig. 1). To assess gibbon habitat quality, we used fruit consumption as an indicator, with higher consumption reflecting higher-quality habitats. A review of fruit consumption of gibbons (Supplementary Table 2) showed that *N. hainanus* had the highest annual fruit intake (85%), indicating a high-quality habitat. In contrast, *N. concolor* (44–81%) and *H. tianxing* (47–49%) inhabit suboptimal habitats with lower and more variable fruit availability, relying more on leaves. Monthly dietary data (Fig. 2B) confirmed these patterns: *N. hainanus* had most stable monthly consumption (range: 39.44%), followed by *N. concolor* (69.01%) and *H. tianxing* (77.58%). *N. hainanus* had the highest fruit consumption of 96.77% in June and remaining above 50% year-round. In contrast, *H. tianxing* had the lowest monthly fruit intake, dropping to 4.82% in February and frequently below 50% in winter. Thus, we classify *N. hainanus* in a high-quality habitat with stable fruit production, while *N. concolor* and *H. tianxing* occupy suboptimal environments with greater seasonal fluctuations.

Further analyses revealed how the gut microbes of different gibbons responded under seasonal and dietary variability. At the ASV level, *N. hainanus* had the lowest alpha diversity (Faith’s phylogenetic index) across the year with a mean value of 22.7 (ranging from 19.4 to 25.6), followed by 26.5 in *H. tianxing* (23–28.7) and 28.9 in *N. concolor* (24.1–34) (Fig. 2C). We further used nestedness to describe the pattern in gut community structures, where species-poor sub-communities are often subsets of species-rich communities. *N. concolor* and *N. hainanus* had higher nestedness than *H. tianxing* (Fig. 2D), with a mean value of 0.05 in *H. tianxing* (0.05–0.06), 0.06 in *N. concolor* (0.03–0.09), 0.07 in *N. hainanus* (0.06–0.09). The higher nestedness observed in *N. concolor* and *N. hainanus* suggested a stable and conserved microbial community structure, likely shaped by the homogeneity of their ecological niche. In contrast, *H. tianxing* showed significantly higher species turnover than *N. concolor* and *N. hainanus* (LMM, all *P* < 0.05, Supplementary Table 3, Fig. 2E). The mean values were 0.75 in *H. tianxing* (0.69–0.78), 0.68 in *N. concolor* (0.62–0.72), and 0.66 in *N. hainanus* (0.64–0.70), indicating the greater variability in gut microbial composition across samples, which could be driven by broader dietary fluctuation. At phylum level, *N. concolor* had the most phyla taxa across the year (Fig. 2F) consistent with the results of Faith’s phylogenetic diversity (Fig. 2C). The gut microbial composition of *N. hainanus* remained relatively stable at both the phylum and ASV levels, while the gut microbial composition of *H. tianxing* is changing from month to month (Fig. 2F–I).

In summary, *N. hainanu*s, inhabiting high-quality habitats, exhibited significantly lower gut bacterial alpha diversity throughout the year and a more stable gut microbial composition compared to the other gibbon species. *H. tianxing*, characterized by strong seasonal dietary variation, showed the lowest nestedness and highest species turnover, reflecting greater gut microbial variability. In contrast, *N. concolor*, with less pronounced seasonal dietary variation, exhibited the highest phylogenetic diversity and included a greater number of phyla (Fig. 2C, I).

### Gut microbial community assembly processes of gibbons with different leaf-feeding levels

**Fig. 3:**
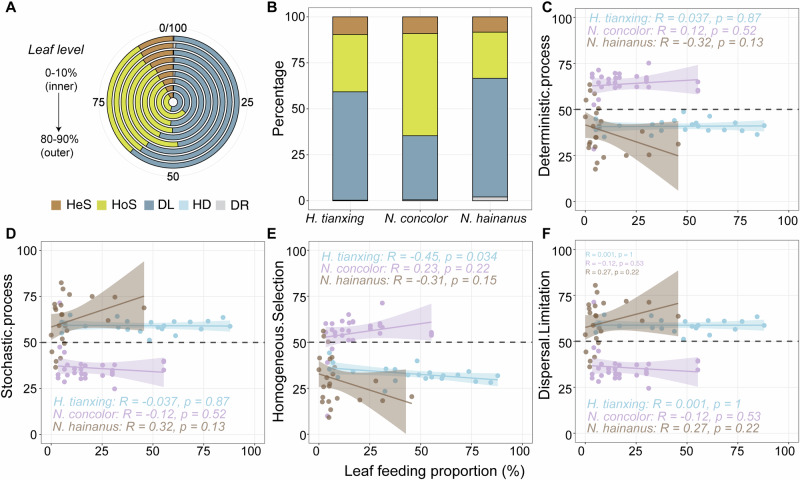
Gut microbial assembly variation linked to leaf feeding in three gibbon species. **A** Microbial assembly processes changing with leaf intake levels. **B** Microbial assembly variation across gibbon species. **C** Deterministic processes linked to leaf consumption. **D** Stochastic processes linked to leaf consumption. **E** Homogeneous selection linked to leaf consumption. **F** Dispersal limitation linked to leaf consumption. HeS heterogeneous selection, HoS homogeneous selection, DL dispersal limitation, HD homogenizing dispersal, DR drift.

To resolve potential mechanisms of microbial community dynamics, we estimated the relative importance of each assembly process for each gibbon species at different leaf-feeding proportion cutoffs (Fig. 3). Among all samples, dispersal limitation was the dominant assembly process across all leave consumption levels (except 10–20%), ranging from ~35 to 61%, followed by homogeneous selection, which ranged from 30 to 56% (Fig. 3A). Specifically, *N. hainanus* exhibited the highest proportion of dispersal limitation at 65%, followed by *H. tianxing* at 60% and *N. concolor* at 35% (Fig. 3B). For *N. concolo*r, homogeneous selection in deterministic processes played a major role (55%, Fig. 3B, C, E and Supplementary Fig. 2). However, the gut microbial community assemblies of *H. tianxing* and *N. hainanus* were dominated by dispersal limitation in stochastic processes (Fig. 3D, F and Supplementary Fig. 2). For *H. tianxing*, homogeneous selection noticeably decreased as the leaf-feeding proportion rose (Spearman’s rank correlation, *P* < 0.05; Fig. 3E).

**Fig. 4:**
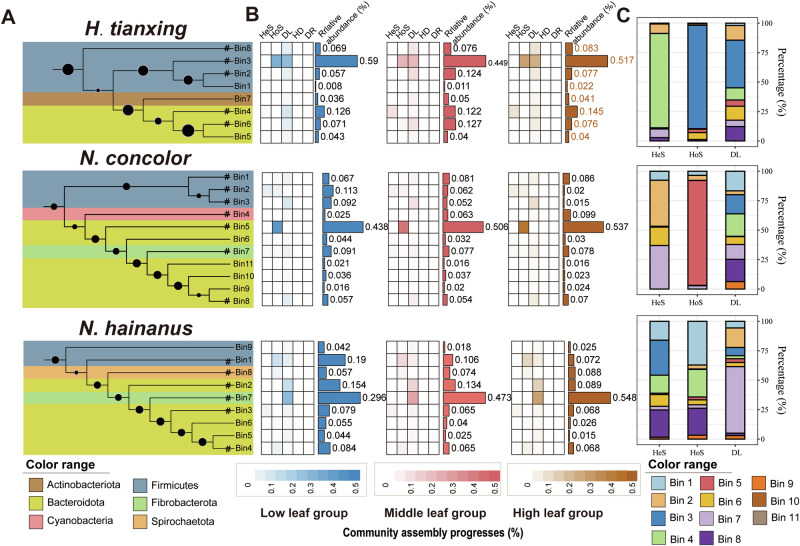
Assembly mechanisms in gut microbial communities of three gibbon species across distinct phylogenetic groupings (bins). **A** Phylogenetic tree overview. The size of dots on the branches of the phylogenetic tree indicates the bootstrap support values, with larger circles representing higher support and confidence. **B** The relative relevance and abundance of several ecological processes in each bin. blue: low leaf-feeding group; pink: middle leaf-feeding group; brown: high leaf-feeding group. For each gibbon species, the leaf-feeding proportion was calculated by per month per family group. All values were sorted in ascending order, with the first 1/3 of the values being the low leaf-feeding group, and so on. The same as below. **C** Relative importance of each bin on gut microbial community assembly. bins: phylogenetic groups, which represent clusters of related organisms based on genetic similarity. HeS heterogeneous selection, HoS homogeneous selection, DL dispersal limitation.

We performed the iCAMP analysis to resolve the potential importance of different bacterial lineages in driving the community assembly processes (Fig. 4). The top 100 ASVs were divided into 8 phylogenetic groups (bins), which represent clusters of related organisms based on genetic similarity, for *H. tianxing*, 11 bins for *N. concolor* and 9 bins for *N. concolor* based on the phylogenetic signal threshold; each was assessed separately for the relative importance of different assembly processes (Fig. 4A and Supplementary Table 4). For *H. tianxing*, Acholeplasmataceae (Bin3) from Firmicutes and Rikenellaceae (Bin4) from Bacteroidota were most important to the total community assembly processes (Fig. 4B and Supplementary Table 5). The importance of Bin4 increased as the leaf proportion rose (Fig. 4B, top panel). The impact of heterogeneous selection, homogeneous selection and dispersal limitation were mainly attributed to the responses of Bacteroidota (>90%), Firmicutes (>90%) and Firmicutes (>70%), respectively (Fig. 4C, top panel). For *N. concolor*, Prevotellaceae from Bacteroidota (Bin5) was most important to the total community assembly processes (Fig. 4B and Supplementary Table 5). The importance of Bin5 to the whole assembly process increased as the leaf proportion rose (Fig. 4B, middle panel). The impact of homogeneous selection was mainly attributed to the responses of Bacteroidota (>95%) (Fig. 4C, middle panel). For *N. hainanus*, Fibrobacteraceae from Fibrobacterota (Bin7) was most important to the total community assembly processes (Fig. 4B and Supplementary Table 5). The impact of dispersal limitation was mainly attributed to the response of Fibrobacterota (>60%) (Fig. 4C, bottom panel). These results highlight the different mechanisms of gut microbial community assembly processes among the three gibbon species.

### The response pattern of gibbon gut microbial communities with different leaf-feeding levels

**Fig. 5:**
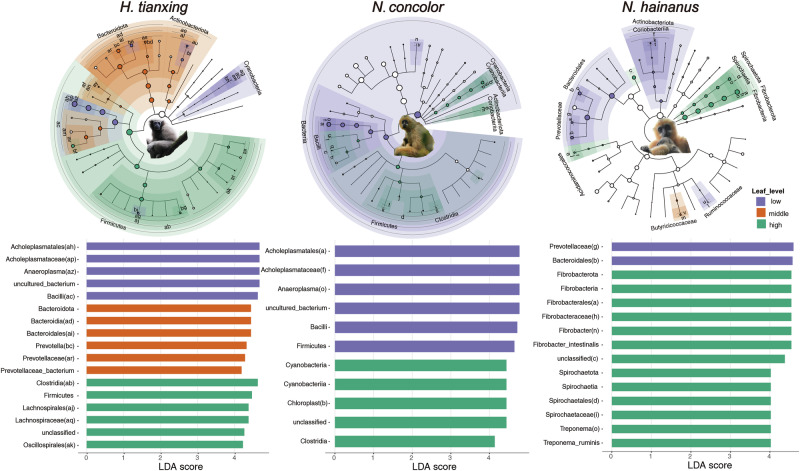
Response bacteria of gibbons with different leaf-feeding proportion. Linear Discriminant Analysis Effect Size (LEfSe) analyses revealed significant contributions to the differences in abundance across various bacteria taxonomic levels, from phylum to genus, among different gibbon species with varying proportions of leaf consumption. Top: The phylogenetic tree shows the hierarchical relationships of microbial taxa, with circle sizes indicating the significance of each taxon. Bottom: The bar plot illustrates the LDA scores of different microbial taxa, highlighting their differential abundance between groups, with longer bars indicating higher significance.

To further explore the potential roles of gut microbial species, we analyzed the relationships between gut microbial communities and leaf-feeding levels in the three gibbons. Using LEfSe analysis (Linear discriminant analysis Effect Size), which identifies biomarkers that differ significantly between groups, we compared the response of gut microbiomes to different levels of leaf consumption. Biomarkers with a Linear Discriminant Analysis (LDA) score of ≥2, indicating a strong association with specific feeding levels, were found in greater numbers in *H. tianxing* (low leaf consumption: 39; middle: 61; high: 94) than in *N. concolor* (low: 12; middle: 1; high: 40) and *N. hainanus* (low: 34; middle: 8; high: 21) (Fig. 5 and Supplementary Table 6).

In *H. tianxing*, Gastranaerophilales (bh) and Anaeroplasma (az) and their respective branches were the main bacteria taxa present in the low leaf-feeding period. In addition, Coriobacteriales (al), Rikenellaceae_RC9_gut_group (bd) and Prevotella (bc) and their respective branches were the main bacteria taxa occurring in the middle leaf-feeding period. Ruminococcaceae (ax), Oscillospiraceae (at), Lachnospiraceae (aq) and their respective branches were the main bacteria taxa present in the high leaf-feeding period. For *N. concolor*, Anaeroplasma (o) and its respective branch were the main bacteria taxa appearing in the low leaf-feeding period. Chloroplast (p), Eggerthellaceae (m) and their respective branches were the main bacteria taxa occurring in the high -eaf feeding period. For *N. hainanus*, Collinsella (t), Prevotellaceae_UCG-001(q), Prevotellaceae_NK3B31_group(s), Alloprevotella (p) and their respective branches were the main bacteria taxa present in the low leaf-feeding period. Treponema (o), Fibrobacter (n), Phascolarctobacterium (r) and their respective branches were the main bacteria taxa found in the high leaf-feeding period. Gastranaerophilales and Chloroplast, both from the phylum of Cyanobacteria, responded to the low leaf-feeding period of *H. tianxing* and the high leaf-feeding period of *N. concolor*. In sum, the three gibbon species exhibited different response taxa under different leaf-feeding levels. More response taxa and phylogenetic branches were detected in *H. tianxing*.

Further, we compared the response taxa at family level among the three gibbon species (Supplementary Fig. 3). We found that some families (e.g., Lachnospiraceae, Oscillospiraceae) exhibited significant positive correlations with an increase in the proportion of leaf feeding across all gibbon species (Supplementary Fig. 3A), suggesting that these microbial families play a key role of the adaptation to a leaf-rich diet. *H. tianxing* and *N. concolor* shared more bacterial families such as Butyricicoccaceae and Ruminococcaceae that significantly increased in relative abundance, as well as bacterial families such as Acholeplasmataceae that significantly decreased in relative abundance as the leaf-feeding proportion rose. For *N. hainanus*, the relative abundance of Fibrobacteraceae, Spirochaetaceae significantly increased, while Coriobacteriaceae, Prevotellaceae and Ruminococcaceae significantly decreased as the leaf-feeding proportion increased (Supplementary Fig. 3A, B). In summary, Lachnospiraceae and Oscillospiraceae were the common response bacterial families for all three gibbons, *H. tianxing* and *N. concolor* shared more response lineages with each other than with *N. hainanus*.

## Discussion

Ecological conservation of wildlife, especially primates, faces many difficulties and challenges, for which new theories and techniques are needed to overcome. Community assembly theories are helpful in understanding the community dynamics of microbes, and are gradually being introduced in the field of microbial ecology. Therefore, this comparative study was conducted to understand intrinsic mechanisms of adaptive strategies among three gibbon species using the theories of community assembly applied to community ecology. In this study, we selected *N. hainanus* from tropical rainforests as a representative of high-quality habitats and *N. concolor* and *H. tianxing* from suboptimal habitats with lower and fluctuating fruit availability. Among the four extant wild gibbon species in China, *N. nasutus* could not be included due to its small and inaccessible population^38^. Our study included all feasible gibbon species to comprehensively examine how habitat differences influence gut microbial communities.

The three gibbon species exhibited different gut bacterial community compositions. Firmicutes and Bacteroidota, the dominant taxa found in the gibbons in this study, are also the prevalent phyla in the gut of many primates^39–41^. Bacteria belonging to Firmicutes and Bacteroidetes are associated with fiber degradation and assist digestion and assimilation of nutrients from plants^42–44^. Proteobacteria and Spirochaetes, two main phyla in captive gibbons, with a total abundance of over 30% of their gut microbial communities^45^, were not considered main phyla of any of the gibbons in our study. Actinobacteriota, the third most abundant phylum in *H. tianxing*, is known to generate secondary antimicrobial compounds^46^ and further mediate gut development. Fibrobacterota, the third most abundant phylum in *N. hainanus*, comprises highly efficient cellulolytic bacteria, important for the degradation of cellulose in the gastrointestinal tracts and best known for its role in rumen function and as a potential source of novel enzymes for bioenergy application^47^. At family level, Prevotellaceae was abundant in all three gibbon species, with the highest abundance in *N. concolor*. These findings are concurrent with previous research, where Prevotellaceae were found to occur in high proportions in the gut of captive *Nomascus* gibbons^45^. Prevotellaceae is associated with diets rich in carbohydrates and high fruit consumption in western lowland gorillas and Verreaux’s sifakas^10,48^. Lachnospiraceae, the third most abundant family in the wild gibbons, is also prevalent in captive gibbons^45^. Lachnospiraceae is an important family in the gut microbial communities of humans and nonhuman primates. Members of Lachnospiraceae specialize in the degradation of complex plant material and are important components of gut health, with their loss resulting in diarrhea^49,50^. Acholeplasmataceae, identified as the most abundant bacterial family in the gut microbiota of *H. tianxing*, is relatively rare in other gibbon species and uncommon among other primates. Members of Acholeplasmataceae are known for their versatility in inhabiting diverse environments, including associations with plant material and soil^51^. reflecting the close ecological interactions of gibbons with their forested habitats. In addition, the ability of Acholeplasmataceae to ferment complex carbohydrates suggests a key role in the digestion of fibrous, plant-rich diets characteristic of gibbons’ ecological niche^52^. These findings highlighted a potential link between Acholeplasmataceae abundance and the dietary habits and ecological adaptations of *H. tianxing*.

Both *H. tianxing* and *N. concolor*, inhabiting suboptimal habitats, had a significantly higher gut microbial alpha diversity than *N. hainanus*, which may offer a distinct set of digestive enzymes to the host, providing benefits to overcome the external environmental pressures^53^. *N. concolor* had the highest bacterial alpha diversity, highlighting that species in microbial communities have great evolutionary differences and may reflect different ecological functions and adaptations^54^. The annual diet of *N. concolor* changes between primarily leaf-based and primarily fruit-based, according to the seasons, (Fig. 2B), requiring different kind of bacteria taxa for digestion (e.g., carbohydrate-degrading and cellulose-degrading bacteria). This result is further confirmed by its highly diverse phylum composition (Fig. 2F). However, *H. tianxing* had significantly higher species turnover than the other gibbons, indicating that the dynamics of gut microbes are more pronounced in primates eating more leaves during a year. When the proportion of leaf feeding increases, many specialized microbes may collaborate with each other for efficient cellulose degradation and energy utilization^55^. This may explain the high number of ASVs found in *H. tianxing*. In *N. concolor* and *N. hainanus*, the diversity of gut microbial communities were also affected by both dietary and seasonal variation, exhibiting lower species turnover (Fig. 2E) and less fluctuation at ASV level (Fig. 2I). These results indicate that frugivorous gibbons have a certain tolerance to the digestion of leaves, and their gut microbiome only becomes unstable when a threshold in the proportion of leaf feeding is reached. However, the gut microbial community of *H. tianxing* was more unstable and volatile, even at the phylum level (Fig. 2F). It is important to monitor the gut microbial dynamic of wildlife, assuming that frequent changes in microorganisms lead to elevated host energy expenditure or increased risks in dysregulation of gut community homeostasis. However, more evidence is needed to support this perspective.

The gut microbial communities of these three gibbon species are all strongly affected by dietary and seasonal variation, which is consistent with many previous studies^4,56,57^. The hindgut of many gibbon species is voluminous and haustrated, with a short, broad cecum^37^, which assists cellulose digestion to a lesser extent than the digestive tract of leaf monkeys. Fruits are easily digestible and require less adaptation to specific microbes^39^. Therefore, frugivorous primates may gradually establish gut microbial communities for leaf digestion, explaining the dynamic community changes with dietary variation. The three gibbon species showed different response patterns to variations of external environmental factors, however.

Microbial patterns in response to high proportions of leaf feeding and the way they influence community structures differ among gibbons. The phyla that dominated the gut microbial assembly of *H. tianxing*, *N. concolor* and *N. hainaus* were Firmicutes, Bacteroidota and Fibrobacterota, respectively (Fig. 4). At the family level, the relative abundance of Acholeplasmataceae was highest in the gut of *H. tianxing*, and was significantly negatively correlated with the proportion of leaves in their diet (the same as in *N. concolor*, Supplementary Fig. 3A). There is limited information on the role of Acholeplasmataceae in the gut, but some studies found that its abundance was positively correlated with the severity of colitis in mice^58,59^. Based on this, we suspect the decline in the relative abundance of Acholeplasmataceae is a good sign for gibbon health. Similar patterns were found regarding a positive effect of Fibrobacteraceae in *N. hainanus*. The family Fibrobacteraceae (phylum: Fibrobacteres) encompassed two cultured species: *Fibrobacter succinogenes* and *Fibrobacter intestinalis*, which are recognized as primary cellulose degraders in the gut systems of ruminants^60,61^. A previous study demonstrated that mature rumen communities exhibit a 570-fold greater abundance of Fibrobacter compared to those in the rumen of preruminant calves, highlighting the critical role of Fibrobacteraceae in the digestion of plant fibers in cattle rumens^62^. This interpretation is supported by the fact that Lachnospiraceae spp., Ruminococcaceae spp. and the family Oscillospiraceae spp. are highly prevalent in cattle and sheep rumen, producing short-chain fatty acids in the cellulolytic processes and harboring various functional CAZymes involved in cellulose and hemicellulose degradation^4,48,63^. The positive response of Lachnospiraceae and Oscillospiraceae to leaf feeding highlights their role in digesting fibrous plant material, which supports gibbons’ dietary flexibility and their ability to adapt to fluctuating ecological conditions. In sum, gibbons differ in their adaptive strategies toward dietary shift and seasonal variation.

We found that different gibbons have different community assembly mechanisms. Gut microbial compositions in *H. tianxing* and *N. hainanus* were dominated mainly by dispersal limitation within stochastic processes (Fig. 3). Previous research has indicated that stochastic processes often dominate when environmental filters are weak, in which case the microbial community would reach a steady state on longer timescales^19^. In the gut system, the microbial community assembly process is strongly influenced by the nutritional composition of host’s diet^64,65^. If dietary changes are not sufficient to drastically change the nutritional profile of the intestinal environment, the assembly processes is likely to be driven by stochastic processes^19^. The diets of *H. tianxing* and *N. hainanus* are primarily leaf-based and fruit-based, respectively. Over long evolutionary timescales, the pressure of environmental filters is too weak for deterministic community assembly, when consuming similar nutrients from the same food items. Consequently, deterministic processes are less impactful on the assembly of microbiota in the gut of *H. tianxing* and *N. hainanus*.

Conversely, deterministic selection may play an increasingly important role as organisms actively shape their environment, such as by depleting resources^19^. Deterministic processes are strongly linked to habitat associations that have likely developed through evolutionary adaptation^66^. *N. concolor*, with a more inter-monthly variable diet (Fig. 2A), would encounter stronger environmental filters, which favors deterministic assembly. Specifically, shifts in dietary structure, such as increased leaf feeding, necessitate specific microbial adaptations for efficient cellulose degradation, increasing selective pressures that influence community composition^67^. As the degradation process of cellulose requires specific types of microbes, it may take some time to establish and maintain populations of these key microbes. As a result, *N. concolor* had the highest alpha diversity of gut microbial communities. In addition, the gut bacterial community assembly of *N. concolor* was driven primarily deterministically rather than stochastically on a short time scale (Fig. 3). Notably, we found that changes in leaf-feeding proportion only significantly altered homogenerous processes of the community assembly of *H. tianxing*, and not the two other gibbons, indicating the variability of gut microbes in *H. tianxing*. Homogeneous selection refers to the selective effects formed by similar abiotic environments and biotic interactions in both time and space, resulting in compositional convergence among microbial communities^68^. In fact, the community assembly of gut microbiota may be more complicated than that of free-living microbes, as gut microbiota are not only affected by the environment but also significantly by host ecology and physiology^69^. While our study emphasizes the role of ecological factors in driving gut microbial assembly, we acknowledge that host genetic background likely influences microbial composition. In addition, the use of amplicon sequencing in this study has inherent limitations in capturing the full functional potential of microbial communities. Future studies employing approaches, such as metagenomics or metatranscriptomics, could provide deeper insights into the functional dynamics of gut microbiota and their relationship to habitat differentiation.

In conclusion, this study revealed the community assembly processes and species turnover of gut bacterial communities in wild primate species, demonstrating the different ecological adaptive mechanisms of three gibbon species. Compared to the other gibbons, we found the species (*H. tianxing*) living in the harshest environment with low temperatures to have the most variable gut microbial community. This study is the first to investigate the ecological adaptive processes in wildlife using microbial community assembly theories, and the results can provide a basis for further comparative studies of primates inhabiting different habitats. Even though gut microbial diversity, response patterns and adaptive strategies differ between gibbons, they seem to be able to cope with short and long-term dietary changes as well as suboptimal habitats, providing hope for conservation efforts.

## Methods

### Study sites and study species

Our research was conducted at three different national nature reserves in China. For the Skywalker hoolock gibbon (*H. tianxing*), we conducted research at two sites within Mt. Gaoligong National Nature Reserve, Yunnan, China: Nankang (24°49′N, 98°46′E) and Banchang (N25°12′, E98°46), respectively. The annual mean temperature was 13.3 °C from October 2010 to September 2011 at Nankang and 13.0 °C from June 2013 to May 2015 at Banchang. The minimum temperature was −2.2 °C in January at Nankang and dropped to −3 °C in December at Banchang^38,70^. Both habitats experience marked seasonal changes, with significant temperature fluctuations between winter and summer, making them the coldest of all habitats in this study.

For the western black crested gibbon (*N. concolor*), we conducted research at the Dazhazi Gibbon Research Station (100°42ʹE, 84 24°21ʹN) in Wuliangshan National Nature Reserve, Yunnan, China. The local mean annual temperature was 15.7 °C, with the lowest mean monthly temperature occurring in January (10.1 °C) and the highest in June (19.2 °C). Annual precipitation was 1793 mm, with a distinct rainy season from May to October (84% of the rainfall)^71^. Therefore, this habitat is also strongly seasonal compared to tropical areas.

For the Hainan gibbon (*N. hainanus*), we conducted research at two sites in the Hainan Tropical Rainforest National Park, Hainan Province, China (18°48′–112 19°12’N, 108°55’–109°17’E). The local area is shaped by tropical monsoon climate with a distinct wet season from May to October and a dry season from November to April^72^. It had an annual average temperature of 21.3 °C and annual precipitation of 1657 mm^73^. Therefore, this habitat is not considered strongly seasonal and the fruit production is higher than in the other habitats.

### Feeding behavior observation and fecal sample collection

The fecal samples and related dietary data of *H. tianxing* used in this study are from a published paper^4^. We used the same protocol for focal behavior observations and sample collections for *N. concolor* and *N. hainanus*. In brief, each month we followed the animals for an average of eight days. Studied animals were located by visiting their sleeping sites of the previous day, listening to their loud calls, and visiting the fruit trees that the studied objects frequented. Once found, studied animals were tracked until they arrived at their sleeping sites. Through continuous observation, the food species and food type, including leaves, fruits, flowers and others (animals, and unknown/unidentified diet items) eaten by the studied animals were recorded by both 5-min-scan and ad libitum methods^74^. The observed time primates spent on specific food items was used to quantify the proportions of each food type instead of directly estimating the amount of food consumed. To compare the fruit consumption among different gibbon species, we conducted a systematic literature search on Web of Science using the keywords “gibbon” and “diet”. In addition, we performed individual searches for each of the 20 gibbon species using their Latin names to retrieve all diet-related publications. This approach ensured that no diet-related studies of all gibbon species were overlooked, providing a comprehensive dataset for our analysis.

To ensure sample quality, fecal samples were collected immediately after defecation (typically less than 5 min). Fecal samples free of soil and litter contamination were collected carefully and aseptically. Samples were placed into 50-ml sterile tubes with 95% ethanol^75^ and then transported to the laboratory, where they were stored at −80 °C prior to subsequent processing. In total, we collected 947 fecal samples: 433 from *H. tianxing*^4^, 234 from *N. concolor* (March 2021 to February 2022), and 280 from *N. hainanus* (January 2022 to December 2022). Detailed information for each sample, including sampling date, family group, age, sex, and other relevant details, is provided in Supplementary Table 7.

### DNA extraction, sequencing, and processing

We used the same protocol for the fecal samples from *N. concolor* and *N. hainanus* for DNA extraction, sequencing and processing as previously described^4^. The V4 hypervariable region of the 16S ribosomal RNA genes was amplified using the primer set F515 (5’-GTGCCAGCMGCCGCGGTAA-3’) and R806 (5’-GGACTACVSGGGTATCTAAT-3’). Sequences were (re)analyzed in QIIME2 (2022.08), and taxonomy was assigned using a Naïve Bayes taxonomy classifier against the SILVA SSU 138.1 database.

### Data visualization and statistical analyses

All analyses and figure productions were performed using R (v 4.3.1). The amplicon sequence variants (ASVs) in the samples among different primates were shown by Venn diagrams using the vennDiagram package (v 1.7.3). The alpha diversity (Faith’s phylogenetic index) was calculated using the vegan package (v 2.6.4). We examined gut bacterial community differences of three gibbons using permutational multivariate analysis of variance (PERMANOVA) based on 999 permutations of a Bray–Curtis distance matrix using the functions “adonis2” for the entire set of ASVs and “pairwiseAdonis2” for the post hoc tests from R packages vegan and pairwiseAdonis (version 0.4). To test for non-random patterns in phylogenetic beta diversity (PBD), the observed PBD between the turnover and the nestedness components of beta diversity were decomposed using the betapart package (v 1.6). We estimated the relative contributions of five different mechanisms, including homogeneous selection, heterogeneous selection, dispersal limitation, homogenizing dispersal, and “drift and others,” in assembling microbial communities according to βNTI (beta Nearest Taxon Index) and RCbray (Bray–Curtis-based Raup Crick metrics)^76^. We used the Phylogenetic-bin-based null model to infer the proportions of these community assembly mechanisms for individual phylogenetic bacterial taxa using iCAMP package (version 1.5.12)^77^ with the parameters ds (phylogenetic signal threshold) = 0.2 and Nmin (minimum number) = 48 in the binning step. We conducted this computation using the “icamp.bins” function. We first conducted iCAMP analysis by pooling samples from the three gibbon species, then we applied the analysis by pooling samples of the leaf level for each gibbon species, and finally, we analyzed each leaf-feeding proportion (per group per month) at each species. Scores for individual processes across all groups were weighted by their relative abundance and aggregated to estimate the relative importance of individual groups at the overall community level. Random forest analysis was used to estimate the importance of influencing factors (family group, individual identity, age, sex, the proportion of both leaf and fruit feeding, longitude, latitude, elevation, month) for faith phylogenetic diversity of gut bacterial community using the rfPermute (v 2.5.1). Only numerical variables with correlation analyses were plotted in the heatmap. We ran linear mixed models (LMM) with post hoc test to compare the differences among species that affect diversity of gut bacterial communities (Faith’s phylogenetic index, turnover, and the nestedness), with individual’s ID and months of sampling as random effects using lme4 package (v 1.1.3). We identified ASVs represented in different leaf-feeding groups using Linear discriminant analysis effect size (LEfSe) by microeco (1.6.0). The threshold of the logarithmic LDA score was set to 2 for discriminative features. We corrected all *P* values using the Benjamini–Hochberg method (BH). Differences with corrected *P* < 0.05 were considered statistically significant.

## Supplementary information

Supplementary figures

Supplementary Tables

## Data Availability

Raw sequence data for bacterial 16S rRNA gene reported in this paper have been deposited (PRJCA027645) in the Genome Sequence Archive in the BIG Data Center, Chinese Academy of Sciences under accession codes CRA017532 and are publicly accessible at http://bigd.big.ac.cn/gsa.

## Code availability

The R scripts and relevant data were provided on GitHub at https://github.com/liying-lan/Gibbon-gut-microbiome.git.
