# Supplementary material for: Comparative study of gut microbiota reveals the adaptive strategies of gibbons living in suboptimal habitats
Source: NPJ Biofilms Microbiomes. 2025 Feb 14;11:29. doi: 10.1038/s41522-025-00653-6 (PMC11828964; doi:10.1038/s41522-025-00653-6)
Supplement: Supplementary file 1 — Supplementary figures [file 41522_2025_653_MOESM1_ESM.pdf]

Supplementary Materials for

Comparative study of gut microbiota reveals the adaptive strategies of gibbons living in suboptimal habitats

Li-Ying Lan[1,2], Tai-Cong Liu[3], Shao-Ming Gao[1], Qi Li[1], Li Yang[1], Han-Lan Fei[4], Xu-Kai Zhong[1], Yu-Xin Wang[1], Chang-Yue Zhu[1], Christoph Abel[2,5], Peter M. Kappeler[2,5], Li-Nan Huang[1]*, Peng-Fei Fan[1]*

***Corresponding author:**

Li-Nan Huang, eseshln@mail.sysu.edu.cn

Peng-Fei Fan, fanpf@mail.sysu.edu.cn

This PDF file includes:

Supplementary Figure 1 to 3

Other Supplementary Materials for this manuscript include the following:

Supplementary Table 1 to 7

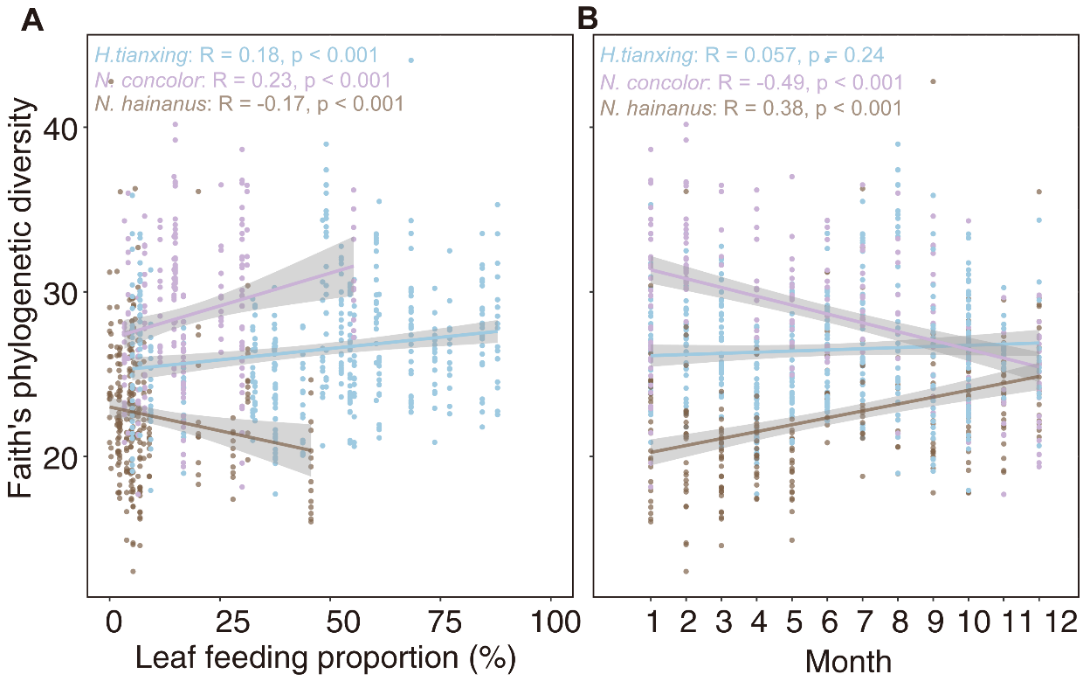

Supplementary Figure 1 | Relationships between gut bacterial alpha diversity and the main drivers

A diet; **B** seasonal variation

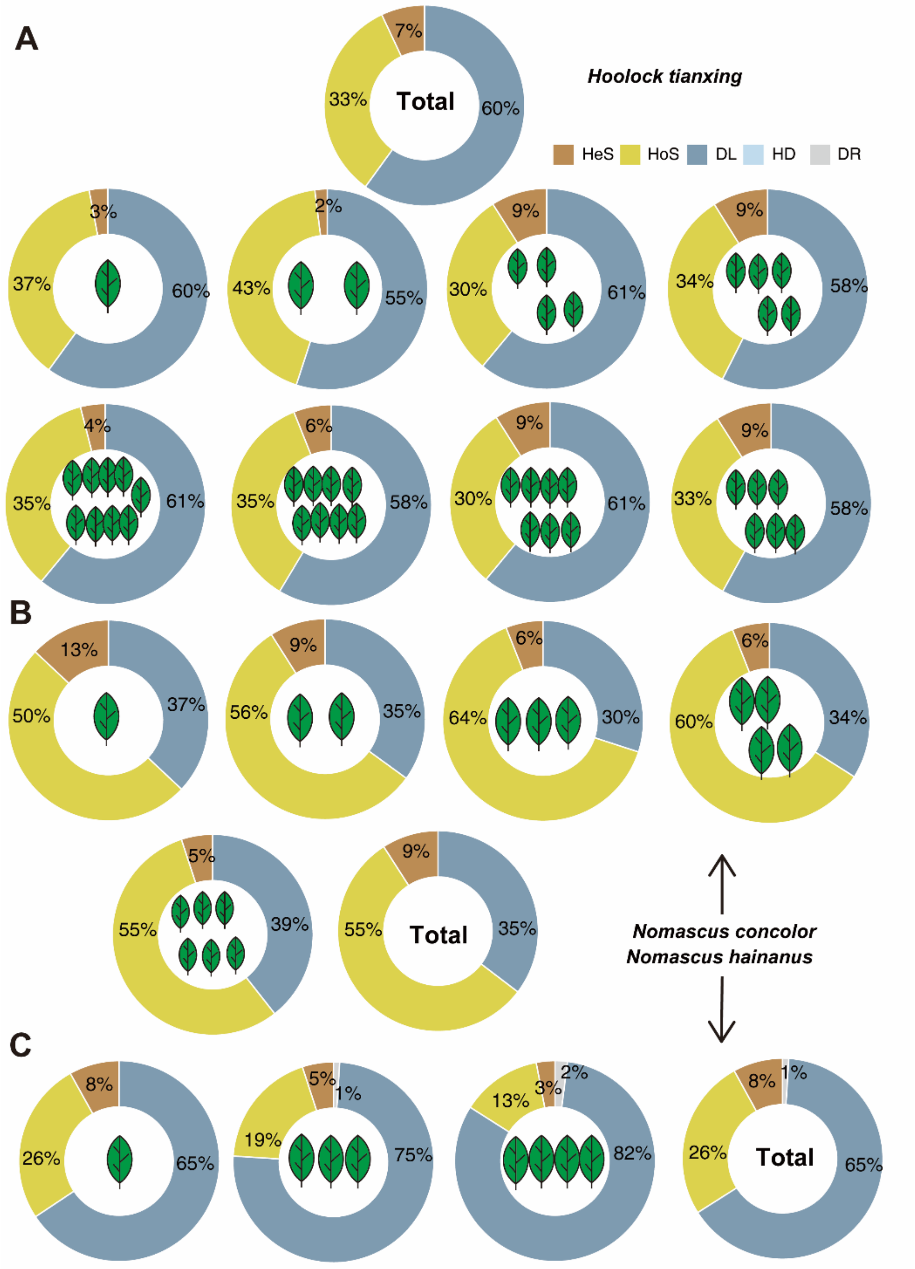

Supplementary Figure 2 | The gut microbial assembly process of three gibbons with different leaf feeding proportions

Note: one leaf represents the leaf feeding proportion is between 0-10%, two leave represents the leaf feeding proportion is between 10-20%, and so on

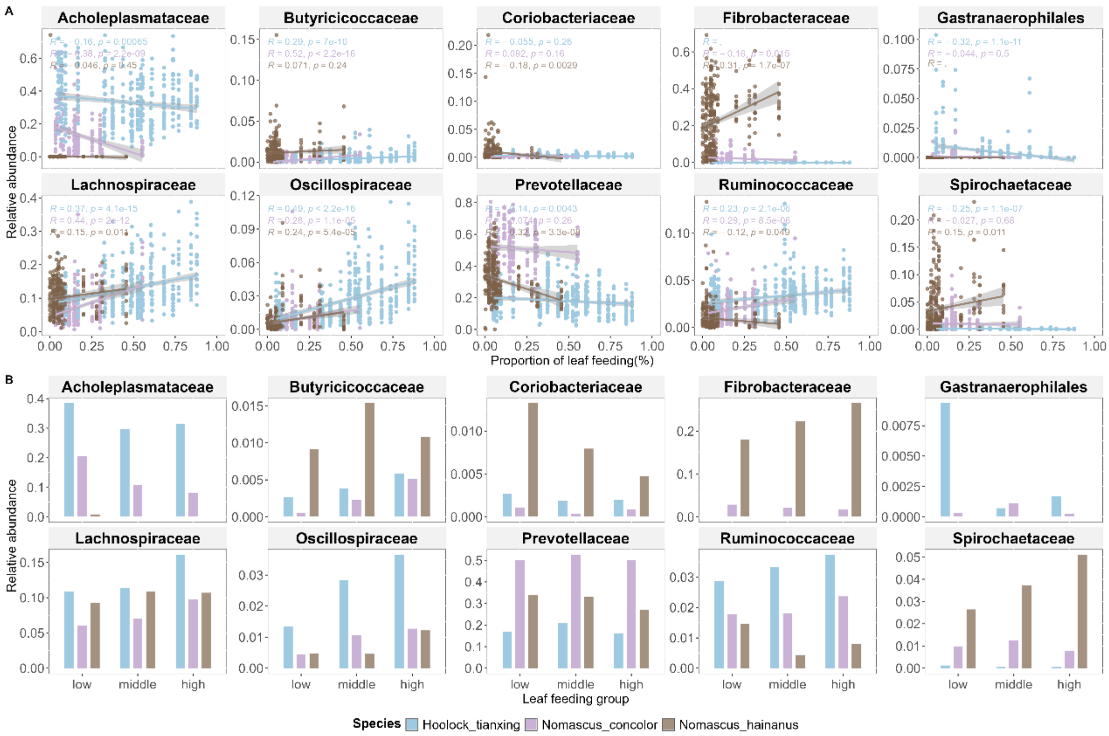

Supplementary Figure 3 | The response patterns of the gut microbial communities at family level along the increasing of the leaf feeding proportions in three gibbons.

Supplementary Table 1 | The role of environmental variation in shaping gut microbial diversity in three gibbon species (Random Forest Model)

Supplementary Table 2 | Feeding information of different gibbon species

Supplementary Table 3 | The post-hoc test of the mixed linear models explaining the species differences on the gut microbial community diversity

Supplementary Table 4 | The importance of each Bin contributes to each community assembly process of gibbons with different leaf feeding level
Bin: phylogenetic group, which represent clusters of related organisms based on genetic similarity

Supplementary Table 5 | Annotation of each Bin with relative abundance

Supplementary Table 6 | Biomarker detected of gibbons with low, milddle and high leaf feeding level (Group) by LEfSe analysis. Source data for Fig5 in the main text

Supplementary Table 7 | Abiotic and biotic data for the 1101 fecal samples from three gibbon species. NA, data not available
